# Pathway Maps of Orphan and Complex Diseases Using an Integrative Computational Approach

**DOI:** 10.1155/2020/4280467

**Published:** 2020-11-27

**Authors:** Kais Ghedira, Soumaya Kouidhi, Yosr Hamdi, Houcemeddine Othman, Sonia Kechaou, Sadri Znaidi, Sghaier Haïtham, Imen Rabhi

**Affiliations:** ^1^Laboratory of Bioinformatics, BioMathematics and Biostatistics (LR16IPT09), Pasteur Institute of Tunisia, University of Tunis El Manar, 1002 Tunis, Tunisia; ^2^Laboratory of Biotechnology and Bio-Geo Resources Valorization (LR11ES31), Higher Institute for Biotechnology, University of Manouba-Biotechpole of Sidi Thabet, 2020, Sidi Thabet, Ariana, Tunisia; ^3^Laboratory of Biomedical Genomics and Oncogenetics, LR16IPT05, Pasteur Institute of Tunis, University of Tunis El Manar, Tunis, Tunisia; ^4^Sydney Brenner Institute for Molecular Bioscience, Faculty of Health Sciences, University of the Witwatersrand, Johannesburg, South Africa; ^5^Laboratoire de Microbiologie Moléculaire, Vaccinologie et développement Biotechnologique, Institut Pasteur de Tunis, University of Tunis El Manar, Tunis, Tunisia; ^6^Laboratory of “Energy and Matter for Development of Nuclear Sciences” (LR16CNSTN02), National Center for Nuclear Sciences and Technology (CNSTN), Sidi Thabet Technopark, Sidi Thabet, Tunisia; ^7^Laboratoire de Parasitologie médicale, biotechnologies et biomolécules, Institut Pasteur de Tunis, University of Tunis El Manar, Tunis, Tunisia; ^8^Higher Institute of Biotechnology at Sidi-Thabet (ISBST), University of Manouba, Tunis, Tunisia

## Abstract

Orphan diseases (ODs) are progressive genetic disorders, which affect a small number of people. The principal fundamental aspects related to these diseases include insufficient knowledge of mechanisms involved in the physiopathology necessary to access correct diagnosis and to develop appropriate healthcare. Unlike ODs, complex diseases (CDs) have been widely studied due to their high incidence and prevalence allowing to understand the underlying mechanisms controlling their physiopathology. Few studies have focused on the relationship between ODs and CDs to identify potential shared pathways and related molecular mechanisms which would allow improving disease diagnosis, prognosis, and treatment. We have performed a computational approach to studying CDs and ODs relationships through (1) connecting diseases to genes based on genes-diseases associations from public databases, (2) connecting ODs and CDs through binary associations based on common associated genes, and (3) linking ODs and CDs to common enriched pathways. Among the most shared significant pathways between ODs and CDs, we found pathways in cancer, p53 signaling, mismatch repair, mTOR signaling, B cell receptor signaling, and apoptosis pathways. Our findings represent a reliable resource that will contribute to identify the relationships between drugs and disease-pathway networks, enabling to optimise patient diagnosis and disease treatment.

## 1. Introduction

Orphan diseases (ODs) or rare diseases are chronic and progressive genetic disorders affecting a small number of people. The US Rare Disease Act of 2002 defined it as a disease that affects fewer than 200,000 inhabitants, equivalent to approximately 6.5 patients per 10,000 inhabitants [1], while in Europe, it is defined as a pathology that affects less than 1 in 2,000 people. To date, between ~6,000 and 8,000 distinct rare diseases have been reported [2]. Patients with ODs are widely heterogeneous with respect to type and complexity of diseases as well as their clinical manifestations and age of onset. Orphan diseases can severely impact patient quality of life and represent a serious burden on society [3]. The principal fundamental aspects related to ODs include insufficient knowledge of physiopathological mechanisms necessary to avoid misdiagnosis and to develop appropriate multidisciplinary healthcare procedures. The second obstacle in ODs strategy management is the lack of knowledge and awareness in medical communities, in addition to the availability of very few patients for clinical trials [4] making these diseases neglected [5]). Unlike ODs, complex diseases (CDs) are with higher incidence and prevalence. Along with the development of high-throughput sequencing technology and population genomics, the scanning for genes or mutations related to complex traits (or diseases) has been greatly promoted. Furthermore, multicohort Genome-Wide Association Studies (GWAS) have currently identified hundreds of genetic variants that are significantly associated with CDs [6]. The availability of biological data from the use of GWAS, massive parallel sequencing, and omics data pushed scientists to use computational approaches in the context of complex biological systems and integrative biology to study disease-disease interactions. Indeed, Goh and Choi [7] have used OMIM data to construct the human diseasome by connecting diseases that share common disease-causing genes. This integrative biology approach is aimed at understanding the relationship between diseases based on the underlying biological mechanisms and is expected to improve our current knowledge of disease cross-talk, which may lead to further improvements in disease treatment. Moreover, DiseaseConnect integrates comprehensive omics, literature data, and drug-related data to reveal disease and disease connectivity via common molecular mechanisms. This tool is very useful since it allows to group diseases with entirely different pathologies, leading to a similar treatment design [8]. It is well known that genes are participating in complex pathways through synergic interactions with other genes, proteins, and environmental factors that collectively influence the clinical manifestations of diseases [9]. A previous study used a computational approach to linking diseases together based on shared pathways [10]. Deciphering molecular pathways of orphan diseases is a key element for understanding molecular events involved in complex disorders [11, 12] and could be investigated as models for the treatment of more complex diseases. In the present analysis, we attempt to investigate the relationship between ODs and CDs using an integrative computational approach based on disease-gene association and gene-pathway association resources to identify potential shared molecular pathways in ODs and CDs and to provide valuable results that can be explored to improve disease diagnosis, prognosis, and treatment.

## 2. Materials and Methods

### 2.1. Gene-Disease Association Integration

Data related to disorders, candidate disease genes, and associations between them were extracted from the Online Mendelian Inheritance in Man [13], DisGeNet [14], and Orphanet [15] databases. The OMIM database, a compendium of human disease genes and phenotypes, is considered to be the best-curated resource of known phenotype-genotype relationships. It was initially focused on monogenic disorders but later on expanded to include complex traits and genetic mutations that confer susceptibility to complex disorders [13]. In addition, the DisGeNet database represents another comprehensive discovery platform designed to address multiple questions regarding the genetics of human diseases. Data in DisGeNet integrates expert-curated information with text-mined data, covering information on Mendelian and complex diseases and all gene-disease associations that include data from animal disease models [14]. Finally, Orphanet represents a unique resource, gathering and improving knowledge on rare diseases so as to improve the diagnosis, care, and treatment of patients with rare diseases [15]. Orphanet is aimed at providing high-quality information on rare diseases and also maintains the Orphanet rare disease nomenclature (ORPHAcode), essential in improving the visibility of rare diseases in health and research information systems (https://www.orpha.net).

### 2.2. Data Harmonization and Processing

Orphanet xml files were collected from the Orphanet portal and parsed to extract disease ID, disease name, and associated genes. From OMIM,we retrieved only phenotype mapping key “(3)” as the molecular basis forthese disorders is known, and mutations have been found in the associated genes. The DisGeNet file was processed, and Unified Modeling Language (UML) identifiers were converted when possible into OMIM ID using OMIM to ontologies file containing the mapping schema in between. Unambiguous UML identifiers corresponding to more than one OMIM ID were discarded. All gene names existing in the three data sources were converted into HGNC official symbols to avoid redundant data. Data collected from the three data sources were harmonized integrated into a single tab-separated file text format. Redundant entries between the distinct data sources (having the same OMIM ID) were removed.

### 2.3. Gene-CD and Gene-OD Network Generation

From integrated data, gene-disease binary links were retrieved. Two bipartite graphs representing OD-gene interactions and CD-gene interactions were constructed using diseases and genes as nodes that have been uploaded into Cytoscape 3.7.2 [16]. A link between a disorder and a disease gene was placed when mutations in that gene lead to that specific disorder.

### 2.4. CD-OD Network Generation

Starting from the gene-disease association bipartite graph, we have generated a new human disease network highlighting biologically relevant CD-OD associations. In this network, the nodes represent the orphan and complex disorders, and two disorders are connected to each other if they share at least one gene in which mutations are associated with both disorders.

### 2.5. Disease-Pathway Mapping

**Figure 1:**
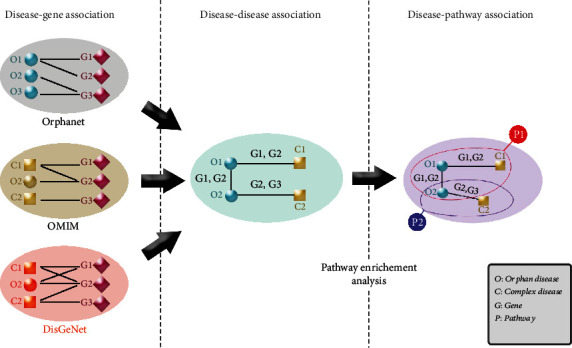
The computational integrative approach undertaken to investigate the disease-gene associations, disease-disease associations, and disease-pathway association.

The Kyoto Encyclopedia of Genes and Genomes (KEGG) pathway enrichment was performed using the Bioconductor package GOstats, Easy Microarray data Analysis package (EMA), False Discovery Rates package (fdrtool), and http://org.Hs.eg.db R packages. We conducted two-sided enrichment/depletion hypergeometric distribution tests in KEGG pathways, with a *p* value significance level of < = 0.05, followed by the Bonferroni adjustment to map pathways to diseases. A Perl script was used to automatically run an R script multiple times on all diseases (https://gist.github.com/radaniba/4170466). Briefly, for each disease in both categories (ODs and CDs), we retrieved the list of associated genes and made a link between the disease and KEGG pathways only if the disease included genes statistically enriched in that pathway.  Figure 1 highlights the integrative approach undertaken here in order to investigate the gene-disease (Orphan and Complex diseases) associations, OD-CD associations, and pathway-human disease mapping.

### 2.6. Networks Analyses

Networks topologies were analysed using the Network Analyzer [16] Cytoscape plugin by computing several metrics including node degree distribution, node eccentricity, node closeness centrality, radiality, stress centrality, and betweenness centrality to identify influential nodes.

## 3. Results

### 3.1. Overlap between Resources and Data Cleansing

**Figure 2:**
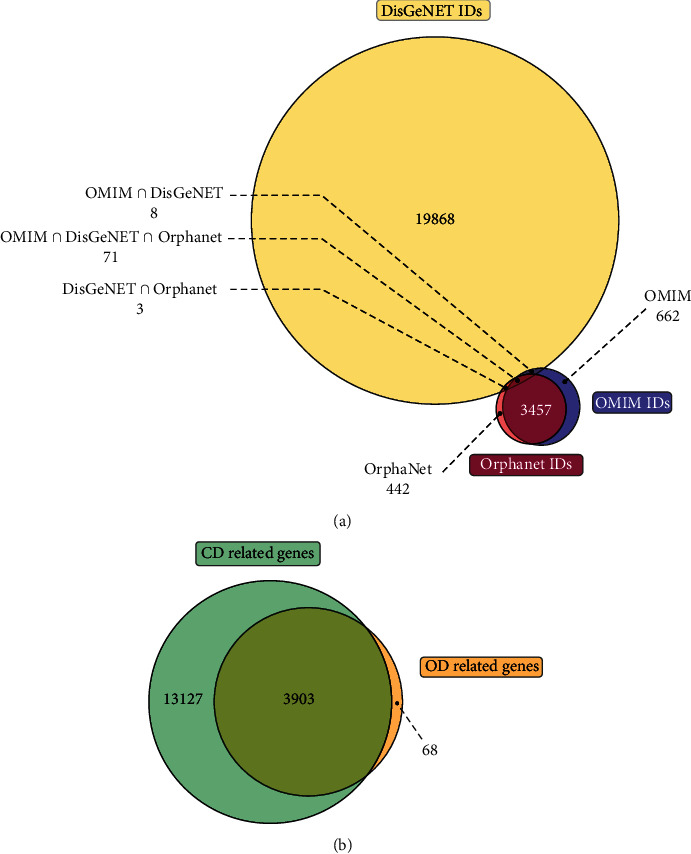
Venn diagrams showing (a) the overlap between diseases in the three data sources used in the present study and (b) the overlap between CD-related genes and OD-related genes.

Unsurprisingly, OMIM and Orphanet resources share a considerable number of overlapping disorders (Figure 2(a)) with 3,528 shared entries between the two databases. This represents about 84% and 88% of the total diseases in OMIM and Orphanet, respectively. On the other hand, the DisGeNet content shared only 0.39% and 0.37% of the total content from OMIM and Orphanet, respectively. All redundant entries between the distinct data sources were removed.

Combined data were processed, and genes associated with diseases were retrieved. In total, we collected 17,030 genes associated with 20,542 diseases and 3,971 genes associated with 3,969 orphan diseases. Finally, we have a total overlap of 3,903 genes between the two groups of genes, related to CDs and ODs (Figure 2(b)).

### 3.2. Gene-Disease Networks

CD-gene associations and OD-gene associations were uploaded into Cytoscape to build gene-disease networks. The OD gene association network is a graph representing 7,748 connected components showing that mainly major diseases are monogenic. The network contains 7,748 nodes representing 3,779 diseases and 3,969 genes with 7,418 edges linking them. Simple parameters of both networks were determined using the Network Analyzer Cytoscape plugin [16] (Table 1). It was previously reported that hub elements within a network are characterized by their high degree of connectivity to other nodes and their central placement in the network [17]. Based on the node degree scores, the OD-gene association network contains many genes that interact with only a few other diseases as well as highly connected genes that interact with many different diseases acting like hubs. Among these hub genes, we identified COL2A1 associated with 19 orphan diseases, LMNA and HBB interacting with 18 ODs, PTEN and FGFR1 linked to 17 ODs, KIT related to 16 distinct ODs, and FGFR3 associated with 15 different ODs (see Supplementary Table 1).

Similarly, when focusing on diseases, we found that some disease nodes interact with a high number of genes including “retinitis pigmentosa” connected to 81 distinct genes and “autosomal recessive nonsyndromic sensorineural deafness type DFNB” related to 73 unique genes.

**Figure 3:**
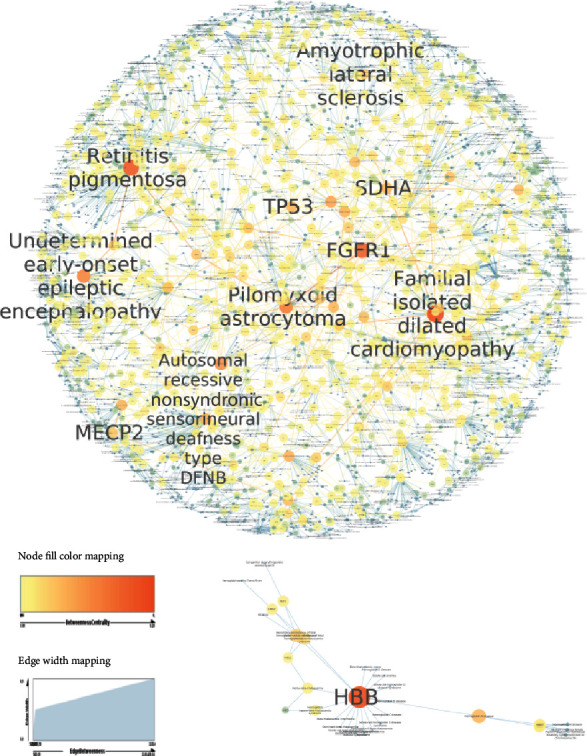
OD-gene association subnetwork. Nodes with orange color denote high influential nodes based on the betweenness centrality measure while those in yellow color denote less central. The network was generated using Cytoscape 3.7.2.

However, to avoid any bias using the node degree centrality score that may favor high studied genes and diseases compared to less studied ones, we identified influential nodes based on the betweenness centrality scores with high values indicating higher relevance of the nodes within the network. Among the most important genes, we report TP53 linked to 14 ODs, FGFR1 linked to 18 ODs, SDHA with 5 links, HBB with 18 links, and MECP2 linked to 7 distinct ODs. We also report “retinitis pigmentosa”, “autosomal recessive nonsyndromic sensorineural deafness type DFNB”, “pilomyxoid astrocytoma” associated with 7 genes, and “familial isolated dilated cardiomyopathy” associated with 44 genes as most central diseases.  Figure 3 highlights these nodes and their interactions with related genes or orphan diseases (Supplementary Table 1).

On the other hand, the CD-gene interaction network represents a compact graph with 60 connected components (Table 1) and characterized by 40,461 nodes representing 17,030 genes and 23,431 diseases with 516,583 edges representing links between them. Based on the betweenness centrality measure, we found that some genes play a central role in the network acting like hubs. This list of genes includes TP53 connected to 1,638 CDs, TNF associated with 1,480 CDs, IL6 involved in 1,189 CDs, BCL2 associated with 891 CDs, IL1B linked to 921 CDs, MTHFR connected to 611 CDs, AKT1 linked to 657 CDs, and VEGFA related to 1,029 CDs.

**Figure 4:**
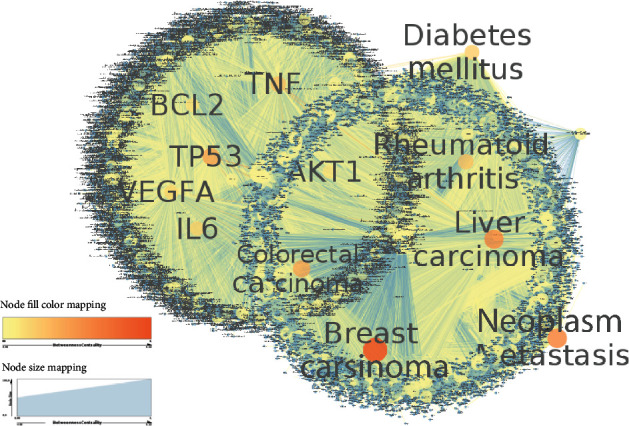
CD-gene association subnetwork. Nodes with orange color denote high influential nodes based on betweenness centrality measure while those in yellow color denote less central. The network was generated using Cytoscape 3.7.2.

Similarly, focusing on diseases, we found that some disease nodes interact with a high number of genes including “breast carcinoma” connected to 4,962 distinct genes, “neoplasm metastasis” linked to 3,913 genes, “colorectal carcinoma” with 2,931 associated genes, “rheumatoid arthritis” with 1,832 linked genes, “diabetes mellitus” with 1,506 gene interactions, and “liver carcinoma” with 3,592 gene associations.  Figure 4 highlights some interactions of these hub nodes with related genes or complex diseases.

### 3.3. Human Disease Network

**Figure 5:**
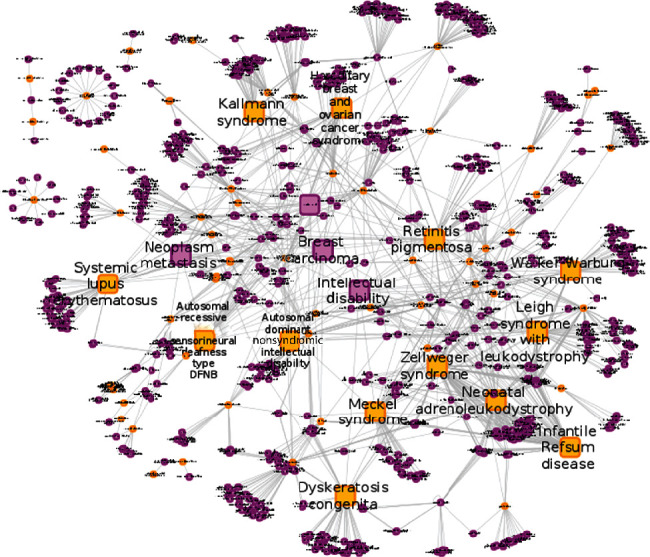
Human diseasome network highlighting the interconnections between ODs and CDs. CD disease nodes are represented in pink colors while OD disease nodes are represented in orange colors. The connection is made between ODs and CDs if both disorders share at least 10 genes.

In order to study the relationships between orphan and complex diseases, we have constructed a human disease network where two diseases are linked together if they share at least one gene based on the disease/gene relationships collected from the different resources [18].  Figure 5 corresponds to the human disease subnetwork including ODs and CDs that present at least 10 genes in common. The network includes 790 nodes corresponding to 76 ODs and 724 CDs linked by 9,378 edges. Linking ODs and CDs in the same network, we found that some ODs including Zellweger syndrome, neonatal adrenoleukodystrophy, the infantile Refsum disease, the retinitis pigmentosa, and the Leigh syndrome with leukodystrophy are linked to several other CDs acting like network hubs. Similarly, we found that intellectual disability, neoplasm metastasis, breast carcinoma, and colorectal cancer act like CD hubs. We also found that rare and more large complex cancers are interconnected within the network through their common genes.

### 3.4. Disease-Pathway Interactions

**Figure 6:**
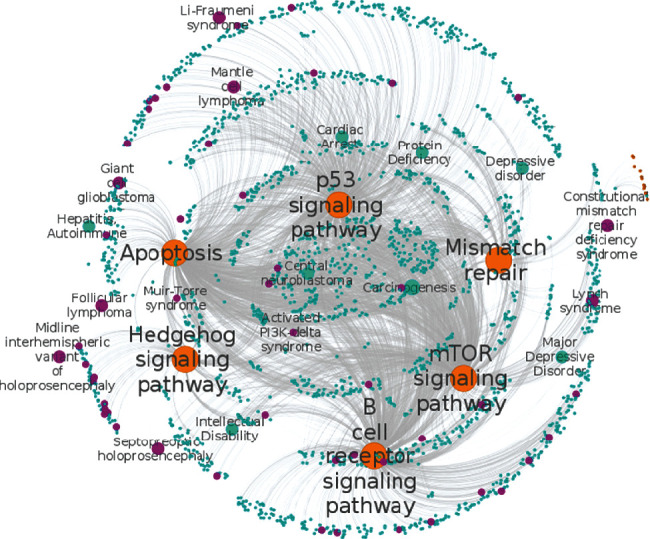
Disease-pathway network highlighting the interconnections between ODs or CDs and pathways. CD disease nodes are represented in cyan color while OD disease nodes are represented in pink color. Pathways are represented in orange color. The connection is made between a disease and a pathway if genes associated with a certain disease are significantly enriched (adjP value ≤ 0.05) in that pathway.

We have initiated this step by identifying human orphan and complex diseases sharing common genes. By linking KEGG pathways to this set of diseases through underlying gene associations and pathway enrichment analysis, we elucidated connections between both types of diseases and metabolic pathways. From the enrichment analysis, we found that only 1,506 out of 3,740 orphan diseases were significantly enriched in KEGG pathways (see Supplementary Table 2). These ODs were linked to 228 different pathways covering a total of 1,256 unique genes. The most significant OD-enriched pathways include focal adhesion pathway (hsa04510), MAPK signaling pathway (hsa04010), regulation of actin cytoskeleton (hsa04810), cytokine-cytokine receptor interaction pathway (hsa04060), and others. We also found that 15,193 out of 24,512 complex diseases were significantly enriched in KEGG pathways (see Supplementary Table 3). These CDs were associated to 229 distinct pathways that involve a total of 4,474 unique genes. The most enriched CD-associated pathways include pathways in cancer (hsa05200), cytokine-cytokine receptor interaction (hsa04060), MAPK signaling pathway (hsa04010), focal adhesion (hsa04510), apoptosis (hsa04210), Toll-like receptor pathway (hsa04620), neurotrophin signaling pathway (hsa04722), and Jak-STAT signaling pathway (hsa04630).  Figure 6 highlights the most enriched common pathways shared by several ODs and CDs. Pathways relative to “cancer,” “apoptosis,” “hedgehog signaling,” “mTOR signaling,” and “mismatch repair” were identified among those shared between ODs and CDs.

The network shows that several rare cancers including hereditary breast and ovarian cancer syndrome and complex cancers including adenocarcinoma and colorectal cancer metastatic are linked through the p53 signaling pathway. This association is expected, as over 50% of human cancers carry the loss of function mutations in the p53 gene [19], suggesting that p53 is a classical Knudson-type tumor suppressor. Moreover, ODs such neurofibromatosis-Noonan syndrome were connected to CDs such as a short neck, coronary artery disease, and intellectual disability through the KEGG MAPK signaling pathway. Neurofibromatosis-Noonan syndrome (NFNS) is a rare condition with clinical features of both neurofibromatosis type 1 (NF1) and Noonan syndrome (NS). It was previously reported that the disease is caused by a dysregulation of the RAS-MAPK pathway through mutations in genes including NF1 and PTPN11 [20]. We also found that the Muir-Torre syndrome which is an autosomal-dominant skin condition of genetic origin, characterized by tumours of the sebaceous gland or keratoacanthoma associated with visceral malignant diseases, is connected to “adenocarcinoma of the colon”, “carcinoma of the lung”, “stomach carcinoma”, “adenocarcinoma of the large intestine”, and other cancers through the KEGG pathway mismatch repair. This association is also expected as it is well established that the most common type of Muir-Torre syndrome is characterized by defects in mismatch repair genes and early-onset tumours [21]. The detailed lists of common pathways associated with ODs and CDs can be found in (Supplementary Table 4).

## 4. Discussion

Several previous studies have used the OMIM database as a unique source of disease-gene association data [13] to study the relations between human diseases. However, these studies may miss information related to certain genes and diseases because OMIM represents a catalogue of inherited Mendelian disorders in man; as a result, most diseases are missed or annotated with very few genes [13, 22]. To compensate for this lack of information related to genes and diseases, we chose to integrate data from the DisGeNet database [14]. This later represents another data source of gene-disease associations integrating information on Mendelian and complex diseases [14]. To study the overlap between complex diseases and orphan diseases, data from the Orphanet database related to orphan diseases were collected and integrated in the present analysis. Orphanet represents a unique resource for gathering and improving knowledge on rare diseases. The database is also developed to refine knowledge about the care and treatment of patients with rare diseases. While this resource is dedicated to orphan diseases, it shares nearly 85% of overlapping disorders with OMIM, and only about 15% are found in the Orphanet database. Using such binary disease gene associations, we found that TP53, FGFR1, and HBB were the top most relevant genes related to ODs (Figure 3). Li-Fraumeni syndrome (LFS) [23] is an inherited autosomal dominant disorder that is usually associated with abnormalities in the tumor suppressor protein P53 gene (TP53) located on chromosome 17p13. LFS variants include LFS1, LFS2, and LFSL. LFS1 is associated with mutations in TP53. Moreover, mutations in the HBB gene are responsible for several serious hemoglobinopathies, including sickle cell anemia and Î^2^-thalassemia. These hemoglobinopathies are a set of hereditary diseases caused by the abnormal structure or insufficient production of hemoglobin [24].

We also found that the TP53, TNF, IL6, VEGFA, and IL1B genes are the top five genes associated with CDs (Figure 4). The TP53 gene encodes for a protein located in the nucleus in which it is able to bind directly to the DNA. When the DNA in a cell becomes damaged by agents such as toxic chemicals, radiation, or ultraviolet (UV) rays from sunlight, this protein plays a critical role in determining whether the DNA will be repaired or the damaged cell will undergo apoptosis if the DNA cannot be repaired. Thus, p53 is essential for regulating DNA repair and cell division. Mutations in the TP53 gene will result in several disorders including breast, colorectal, bladder, lung, and ovarian cancers but also some rare cancers such as the Li-Fraumeni syndrome [25]. These aforementioned relevant genes may be used as biomarkers for drug targets and/or disease diagnostics. A previous study has suggested TP53 as biomarkers of carcinogen exposure and cancer risk and prognosis [26]. We have compared this list of relevant genes with a list of druggable genes from the human protein Atlas https://www.proteinatlas.org/search/protein_class%3AFDA+approved+drug+targets?format=tsv and found that nearly all relevant reported genes belong to this list including FGFR1, SDHA, HBB, TP53, TNF, IL6, BCL2, IL1B, and VEGFA.

We also showed that ODs and CDs can be clustered together through many shared genes such as the TP53 gene. Previously, the Centre for Therapeutic Target Validation was able to develop an experimental factor ontology (EFO) by integrating rare and complex disease-related phenotype and genotype ontologies and identified 20 common diseases and 85 rare diseases that share similar phenotypes [27]. Through Figure 5, we showed that some ODs and CDs could share more than 10 common genes. These shared genes provide the potential possibility for repurposing drugs that have been initially designed for CD to OD therapy and reciprocally [28]. Computational drug repositioning bears a rapid alternative for generating a list of repositioning candidate drugs. The main challenge consists of the experimental verification of the efficacy and safety of these computationally identified candidate drugs and to move them forward into clinical trials, something that remains hard especially with rare disease patients [28]. We finally connected CDs and ODs to biological pathways when the genes associated with these diseases are enriched in common pathways. Interestingly, our results show that apoptosis, mismatch repair, hedgehog signaling, mTOR signaling, B cell receptor signaling, and p53 signaling pathways are significantly enriched in several CDs including many cancers, heart diseases, and diabetes as well as in ODs (Figure 6). To assess the reliability of the present approach, we compared it to prior computational works that focused on breast cancer-related disorders (Wu and coworkers (2008)). Both studies using distinct approaches reported the same list of genes including BRCA1, BRCA2, TP53, PIK3CA, CHEK2, PTEN, ATM, RAD51, PPM1D, and CASP8 as most relevant genes associated with breast cancer. Similarly, as novel breast cancer susceptibility genes, we found AKT1, ESR2, and RAD50 as the top genes. Moreover, we also compared our results to the pathway enrichment among the top 100 breast cancer-related genes and similarly found that the p53 signaling pathway and cell cycle are the most enriched pathways and that our study is in agreement with prior studies that use other approaches.

Besides, previous human disease networks were represented as nodes corresponding to disorders (without differentiating between ODs and CDs) connected to each other if they share at least one gene in which mutations are associated with these disorders [18]. Furthermore, the gene set enrichment analysis is known as a method of choice to study and investigate pathways related to a set of genes [29]. In the present study, we are combining both approaches to investigate pathway-human disease relationships. Indeed, in order to share common pathways, diseases must share some common genes (with mutations) and must be enriched in similar pathways (with adjP − value ≤ 0.05). Through the present approach, we were up to identify novel disease connections through disease-disease and disease-pathway associations that are difficult to detect through a single-gene analysis-based method. Understanding how human diseases and specifically how CDs and ODs are related to each other will bestow potentially new insights into the design of novel pathway-guided therapeutic interventions for human diseases [30]. This study is in line with the novel approaches for biomarker discovery that is switching from the focus on single genes to multiple genes that interact in a cell [31, 32]. It was previously reported [33] that a network-based computational analysis can enhance the efficiency of the drug development process. Moreover, [34] proposed a computational approach that finds drug targets by clustering networks through heterogeneous biomedical data that include genes, biological processes, pathways, and phenotypes.

The ultimate rationale behind this kind of study is that if a pathway is shared by multiple CDs and ODs, a drug being used to treat one disorder could potentially be reused to treat another disorder targeting the same pathway [10]. For example, some of the most effective treatments for coronary artery disease, a complex disease, were first established during the study of familial hypercholesterolemia, an orphan disease [1]. Preliminary computational investigation using the TTD database [35] showed that some existing drugs can be used for both complex and orphan diseases. Indeed, it is well known that the ataluren can be used to treat complex diseases including muscular dystrophy and cystic fibrosis as well as ODs including mucopolysaccharidosis and Dravet syndrome. Baclofen can also be used for multiple sclerosis but also for fragile X syndrome. Bis-choline tetrathiomolybdate was also found to be effective against prostate cancer thanks to its antiangiogenic activity [36]. The component is currently under investigation to confirm its efficacy against Wilson's rare disease characterized by an abnormal accumulation of copper in the body. Tocotrienols are known to target and inhibit the cyclooxygenase-2 (COX-2), a proinflammatory enzyme which is activated in gastric, hepatocellular, esophageal, pancreatic, colorectal, breast, bladder, cervical, endometrial, skin, and lung cancers and is involved in promoting cell survival, angiogenesis, and metastasis [37–39]. An open-label study of the alpha-tocotrienol quinone therapy in 10 children with genetically confirmed Leigh syndrome caused by pathogenic variants in a number of different genes showed stabilization and even reversal of disease progression [40]. Similar integrative approaches may help to provide more complete pictures on the disease relationships and drugs that can be used to target common pathways and treat different diseases.

## 5. Conclusions

In the present study, we have investigated the overlap between CDs and ODs based on the data available from three distinct databases OMIM, DisGeNet, and Orphanet. To achieve our aim, we have taken an integrative computational approach to generate a human gene-disease network and to further study the relationship between human diseases considering the genes that are associated with these diseases and elucidate the relationship between diseases and pathways. Our results showed that an important number of common genes and metabolic pathways are shared between the two types of diseases. The present work provides a resource of exceptional interest that will contribute to identify the relationships between drugs and disease-pathway networks, enabling to optimise patient diagnosis and disease treatment.

## Figures and Tables

**Table 1:** Simple parameters of both networks determined using the network analyzer Cytoscape plugin.

	CDs-gene association network	ODs-gene association network
Number of nodes	40,461	7,748
Number of edges	516,883	7,418
Clustering coefficient	0	0
Connected components	60	1,492
Network diameter	10	34
Shortest paths	1,627,436,746	13,223,970
Characteristic path length	3.717	10.169
Avg. number of neighbors	25.534	1.915
Network density	0.001	0
Network heterogeneity	3.852	1.400

## Data Availability

All data are available as supplementary materials and will be available online with the manuscript.

## Supplementary Materials

Supplementary 1 Supplementary Table 1: Examples of Orphan Diseases associated to hub genes based on degree centrality measure.

Supplementary 2 Supplementary Table 2: Orphan Diseases KEGG pathways enrichment.

Supplementary 3 Supplementary Table 3: Complex Diseases KEGG pathways enrichment.

Supplementary 4 Supplementary Table 4: Orphan Diseases and Complex Diseases Common pathways.
